# A facile iodine(III)-mediated synthesis of 3-(3-aryl-1-phenyl-1*H*-pyrazol-4-yl)-[1,2,4]triazolo[4,3-a]pyridines *via *oxidation of 2-((3-aryl-1-phenyl-1*H*-pyrazol-4-yl)methylene)-1-(pyridin-2-yl)hydrazines and their antimicrobial evaluations

**DOI:** 10.1186/2191-2858-1-1

**Published:** 2011-07-18

**Authors:** Om Prakash, Khalid Hussain, Deepak K Aneja, Chetan Sharma, Kamal R Aneja

**Affiliations:** 1 Institute of Pharmaceutical Sciences, Kurukshetra University, Kurukshetra 136119, Haryana, India; 2 Department of Chemistry, Kurukshetra University, Kurukshetra 136119, Haryana, India; 3 Department of Microbiology, Kurukshetra University, Kurukshetra 136119, Haryana, India

**Keywords:** hypervalent iodine, antimicrobial activity, triazoles, pyrazole

## Abstract

**Background:**

Fused heterocyclic 1,2,4-triazoles have acquired much importance because of their interesting biological properties. Although a number of methods have been reported in the literature which includes oxidation with phosphorus oxychloride, lead tetraacetate, bromine, etc., hypervalent iodine reagents have emerged as reagents of choice for various synthetically useful transformations due to their low toxicity, ready availability and ease of handling.

**Results:**

A series of new 3-(3-aryl-1-phenyl-*1H*-pyrazol-4-yl)-[1,2,4]triazolo[4,3-a]pyridines **4 **has been conveniently synthesized by oxidative cyclization of 2-(3-aryl-1-phenyl-1*H*-pyrazol-4-yl)methylene)-1-(pyridin-2-yl)hydrazines **3 **promoted with iodobenzene diacetate under mild conditions (up to 90% isolated yields). All the new compounds were tested *in vitro *for their antimicrobial activity.

**Conclusions:**

Iodine(III)-mediated oxidative approach has offered an easy access to new 3-(3-aryl-1-phenyl-1*H*-pyrazol-4-yl)-[1,2,4]triazolo[4,3-a]pyridines **4**. The antibacterial and antifungal activities of newly synthesized compounds have proved them potent antimicrobial agents.

## Background

Fused heterocyclic 1,2,4-triazoles have acquired much importance because of their CNS depressant [1], antiallergy [2], antimicrobial [3] and anti-inflammatory [4] properties. Most methods for the preparation of fused 1,2,4-triazole derivatives are based on the oxidation of heterocyclic hydrazones or hydrazides with phosphorus oxychloride [5], lead tetraacetate [5,6], bromine [6,7], etc., which are associated with toxic properties. Therefore, alternative approach avoiding these reagents is always preferred.

Organohypervalent iodine reagents have emerged as reagents of choice for various synthetically useful transformations due to their low toxicity, ready availability and ease of handling [8-17]. We have recently reported the usefulness of iodobenzene diacetate (IBD) to effect oxidative cyclization of benzalhydrazones to 1,2,4-triazoles [18-22].

Pyrazoles form an integral part of many natural products of therapeutic importance and possess potentially reactive sites for a variety of chemical reactions to generate molecular diversity. (*S*)-3-Pyrazolylalanine [23], lonazolac [24], difenamizole [25], mepirizole [26], metamizol [27] and 4,5-dihydro-3-phenyl-6*H*-pyrrolo[1,2-b]pyrazole are some of the biologically active compounds endowed with antimicrobial [28], hypoglycaemic [29] and non-nucleoside HIV-1 reverse transcriptase inhibitor properties [30].

Our ongoing programme on the development of hypervalent iodine-mediated methodologies in heterocyclic synthesis coupled with the significant biological importance of fused 1,2,4-triazole derivatives and pyrazole derivatives, prompted us to undertake the synthesis of hitherto unknown fused 1,2,4-triazolopyridines. We report in this study on the synthesis of fused 3-(3-aryl-1-phenyl-1*H*-pyrazol-4-yl)-[1,2,4]triazolo[4,3-a]pyridines **4 **by the oxidation of 2-((3-aryl)-1-phenyl-1*H*-pyrazol-4-yl)methylene)-1-(pyridin-2-yl)hydrazines **3 **using IBD in dichloromethane with an expectation to find new and more potent antibacterial and antifungal agents.

## Results and discussion

### Chemistry

**Scheme 1:**
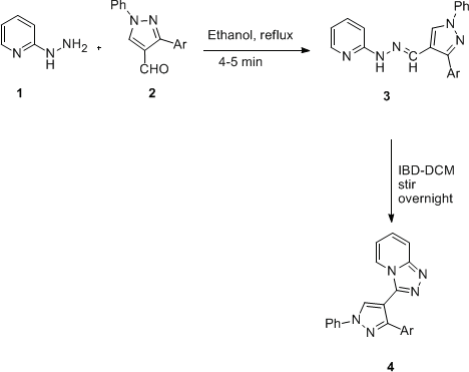
**Synthesis of title compound 4 *via *oxidation of 3**. Ar: Phenyl **(a)**; 4-methylphenyl **(b)**; 4-methoxyphenyl **(c)**; 4-fluorophenyl **(d)**; 4-chlorophenyl **(e)**; 4-bromophenyl **(f)**; 4-nitrophenyl **(g)**.

First, we synthesized a series of 2-((3-aryl-1-phenyl-1*H*-pyrazol-4-yl)methylene)-1-(pyridin-2-yl)hydrazines **3 **needed for their oxidative cyclization. These substrates were easily accessible in high yields (88-96%) and purity from the reaction of 2-pyridyllhydrazine **1 **and 3-aryl-1-phenyl-1*H*-pyrazole-4-carbaldehydes **2 **in ethanol (Scheme 1) [31]. Then, the reaction of 2-((1,3-diphenyl-1*H*-pyrazol-4-yl)methylene)-1-(pyridin-2-yl)hydrazine **(3a)** (see Additional file 1) was carried out with 1.1 equivalents of IBD in dichloromethane by stirring at room temperature overnight. The usual work-up of the reaction afforded the expected product, 3-(1,3-diphenyl-1*H*-pyrazol-4-yl)-[1,2,4]triazolo[4,3-a]pyridine **(4a)** (see Additional file 2) in 90% yield (Scheme 1). To study the scope of reaction, we carried out oxidation of a wide range of substituted 2-((3-aryl-1-phenyl-1*H*-pyrazol-4-yl)methylene)-1-(pyridin-2-yl)hydrazines **(3b-g)** (see Additional files 3, 4, 5, 6, 7 and 8) under similar conditions. It was observed that IBD-mediated oxidative approach worked nicely to give the desired products **4b-g** (see Additional files 9, 10, 11, 12, 13 and 14) in all cases in 82-90% yields.

The structures of all the compounds **3 **and **4 **were confirmed by their spectral (IR, ^1^H NMR, Mass) and elemental analytical data. For example, the IR spectrum of the compound **3a **exhibited characteristic absorption band at 3190 cm^-1 ^due to NH functional group. The ^1^H NMR spectrum of the product **3a **showed two singlets due to C(5)-H of pyrazole ring and N=CH at δ 8.93 and δ 8.17, respectively, and also a broad singlet due to NH at δ 10.66 which disappeared on the addition of D_2_O. Other protons appeared as multiplet in the aromatic regions. Mass spectrum of the compound **3a **exhibited molecular ion peak at *m/z *340.06 [M + 1]^+^.

**Scheme 2:**
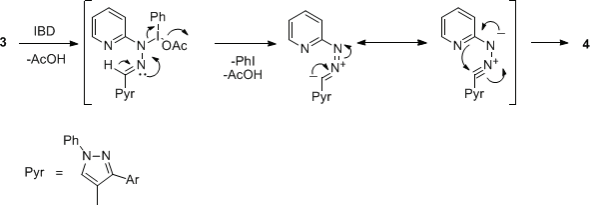
**Mechanism for the oxidation of 3 to 4**.

The characterization of products **4 **was based upon a careful comparison of their IR and ^1^H NMR spectra with those of **3**. IR spectra of **4 **were found to be transparent in the region of NH stretch, thus confirming the oxidation of **3 **into **4**. An important characteristic feature in the ^1^H NMR spectra of **4 **was the disappearance of the singlet due to N=CH around δ 8.12-8.93, which was present in the spectra of **3**. The plausible mechanism for the oxidation of **3 **to **4 **is analogous to our earlier reports [18,19] and given in Scheme 2.

### Pharmacology

#### *In vitro *antibacterial activity

**Figure 1:**
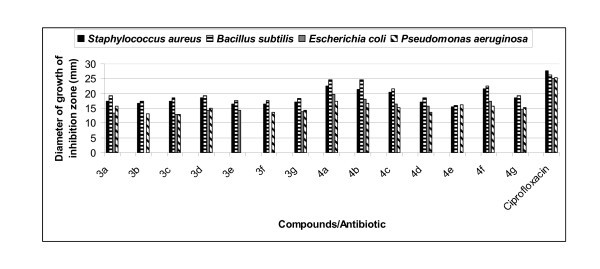
**Comparison of diameter of growth of inhibition of compounds (3 and 4) and standard drug**.

**Figure 2:**
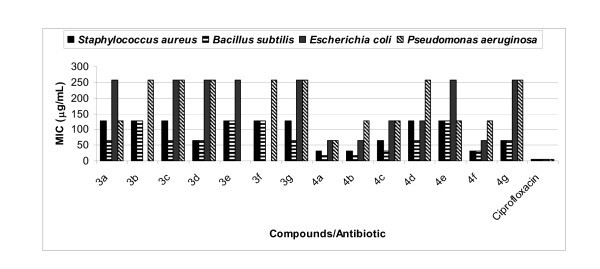
**Comparison of MIC of compounds (3 and 4) and standard drug**.

All the synthesized compounds, **3a-g **and **4a-g **were evaluated *in vitro *for their antibacterial activity against two Gram-positive bacterial strains, *Staphylococcus aureus *and *Bacillus subtilis *and two Gram-negative bacteria namely, *Escherichia coli *and *Pseudomonas aeruginosa*, and their activity was compared to a well-known commercial antibiotic, ciprofloxacin. All the compounds possessed variable antibacterial activity against Gram-positive bacteria, *S. aureus *and *B. subtilis*. Results of antibacterial evaluation are summarized in Table 1 and Figure 1. Compounds **3a-g **and **4a-g **showed zone of inhibition ranging between 12.8 and 24.6 mm. On the basis of zone of the inhibition produced against the test bacteria, compound **3d **was found to be the most effective against *S. aureus *and *B. subtilis *showing the maximum zone of inhibition of 18.6 and 19.3 mm, respectively, when compared with commercial antibiotic ciprofloxacin, which showed maximum zone of inhibition of 27.6 and 26.3 mm against Gram-positive bacteria, *S. aureus *and *B. subtilis*, respectively. Compounds **4a**, **4b**, **4c **and **4f **were found to be the most effective against both Gram-positive bacteria showing maximum zone of inhibition ranging between 20.3 and 22.6 mm. Rest of compounds showed fair activity against Gram-positive bacterial strains (Table 1, Figure 1). All the synthesized compounds showed fair activity against both Gram-negative bacterial strains. In the whole series, the MIC value of various synthesized compounds (**3 **and **4**) ranges between 16 and 256 μg/mL against Gram-positive and Gram-negative bacteria (Table 2, Figure 2). Out of compounds **3a-3g**, compound **3d **was found to be the most effective against both Gram-positive bacteria having the lowest MIC value 64 μg/mL when compared with commercial antibiotic ciprofloxacin, which showed MIC value 5 μg/mL for both Gram-positive bacteria. Out of compounds **4a-4g**, compounds **4a**, **4b, 4c **and **4f **possessed good antibacterial activity against *B. subtilis *with MIC of 16, 16, 32 and 32 μg/mL, respectively (Table 2, Figure 2).

**Table 1:** *In vitro *antibacterial activity of compounds 3 and 4

Compounds	Diameter of growth of inhibition zone (mm)^a^			
	
	*Staphylococcus aureus*	*Bacillus subtilis*	*Escherichia coli*	*Pseudomonas aeruginosa*
**3a**	17.3	19.3	13.3	15.6

**3b**	16.6	17.3	-	13.2

**3c**	17.3	18.6	13.0	12.8

**3d**	18.6	19.3	14.3	15.0

**3e**	16.3	17.6	14.2	-

**3f**	16.3	17.6	-	13.5

**3g**	17.0	18.3	13.8	14.3

**4a**	22.6	24.6	19.6	17.3

**4b**	21.3	24.6	18.0	16.6

**4c**	20.3	21.6	16.3	15.3

**4d**	17.0	18.6	15.6	13.6

**4e**	15.5	16.0	-	16.2

**4f**	21.6	22.6	17.3	15.6

**4g**	18.6	19.3	14.6	15.3

Ciprofloxacin	27.6	26.3	25.0	25.3

-, no activity

^a^Values including diameter of the well (8 mm), are means of three replicates

**Table 2:** Minimum inhibitory concentration (μg/mL) of compounds 3 and 4

Compounds	Minimum inhibitory concentration (μg/mL)			
	
	*Staphylococcus aureus*	*Bacillus subtilis*	*Escherichia coli*	*Pseudomonas aeruginosa*
**3a**	128	64	256	128

**3b**	128	128	NT	256

**3c**	128	64	256	256

**3d**	64	64	256	256

**3e**	128	128	256	NT

**3f**	128	128	NT	256

**3g**	128	64	256	256

**4a**	32	16	64	64

**4b**	32	16	64	128

**4c**	64	32	128	128

**4d**	128	64	128	256

**4e**	128	128	256	128

**4f**	32	32	64	128

**4g**	64	64	256	256

Ciprofloxacin	5	5	5	5

NT, not tested.

#### *In vitro *antifungal activity

**Figure 3:**
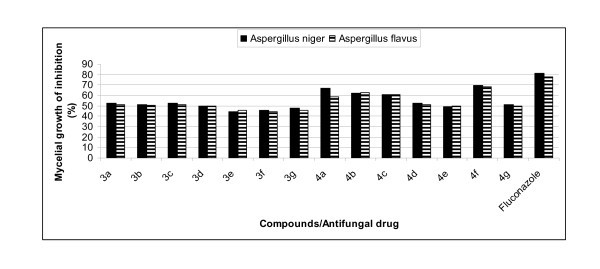
**Comparison of antifungal activity of compounds (3 and 4) and standard drug**.

All the newly synthesized compounds **(3 **and **4) **were also tested *in vitro *for their antifungal activity against two fungi, namely *Aspergillus niger *and *Aspergillus flavus*. Standard antibiotic fluconazole was used for comparison with antifungal activity shown by compounds **3a-g **and **4a-g**. A careful analysis of percentage mycelial growth inhibition revealed that compounds **3a**, **3b**, **3c **and **3d **exhibit good antifungal activity against both *A. flavus *and *A. niger*. Out of compounds **4a-g**, compounds **4a**, **4b**, **4c **and **4f **showed excellent activity against both antifungal strains as shown in Table 3 and Figure 3. It indicates that fused triazoles **4a-g **containing electron-releasing substituents **(4a**, **4b **and **4c) **at *para *position of aryl ring of pyrazole moiety are more antifungal than triazoles having electron-withdrawing groups (**4d**, **4e**, **4f **and **4g**) at the same position.

**Table 3:** *In vitro *antifungal activity of compounds 3 and 4

Compounds	Mycelial growth of inhibition (%)	
	
	*Aspergillus niger*	*Aspergillus flavus*
**3a**	52.5	51.1

**3b**	51.1	50.6

**3c**	52.5	51.1

**3d**	50.0	49.8

**3e**	44.4	45.5

**3f**	45.5	44.4

**3g**	47.7	45.5

**4a**	66.5	58.8

**4b**	62.3	62.5

**4c**	60.5	60.6

**4d**	52.5	51.1

**4e**	48.8	50.0

**4f**	69.5	68.5

**4g**	51.1	50.0

Fluconazole	81.1	77.7

A careful analysis of MIC data revealed some interesting results **(**Table 2) which are as follows

(i) Compounds **3a-g **have shown marginal activity, but after oxidative cyclization of these compounds, it was found that antibacterial activity has been increased.

(ii) Inhibitory data of compounds **4a-g **suggested that the replacement of *para *proton of aryl ring of pyrazole moiety (at 3-position) in 3-(3-aryl-1-phenyl-*1H*-pyrazol-4-yl)-[1,2,4]triazolo[4,3-a]pyridines (**4a-g) **with electron-releasing groups the antibacterial activity increases while replacing the same aryl proton with electron-withdrawing group the antibacterial activity decreases. In compound **4b**, the proton at *para *position of aryl ring of pyrazole moiety is replaced with methyl group, and in compound **4c**, the proton at *para *position of aryl ring of pyrazole moiety is replaced with methoxy group, both of these compounds exhibited significant level of antibacterial activity. Compounds **4d**, **4e**, **4f **(containing halogen at *para *position of aryl ring of pyrazole moiety) and **4g **(containing nitro group at *para *position of aryl ring of pyrazole moiety) have shown marginal activities against all four bacteria.

## Conclusions

We have described in this study an efficient and convenient synthesis of some new 3-(3-aryl-1-phenyl-1*H*-pyrazol-4-yl)-[1,2,4]triazolo[4,3-a]pyridines **(4a-g) ***via *the oxidative cyclization of hydrazones **3**, thereby emphasizing the increasing utility of hypervalent iodine(III)-mediated methods. The antibacterial and antifungal activities of newly synthesized compounds **3 **and **4 **have proven them potent antibacterial and antifungal agents.

Compounds **3a-3g **have shown marginal activity. Inhibitory data of compounds **4a-4g **suggested that by the replacement of *para *proton of aryl ring of pyrazole moiety in 3-(3-aryl-1-phenyl-1*H*-pyrazol-4-yl)-[1,2,4]triazolo[4,3-a]pyridines (**4a-g) **with electron-releasing groups the antibacterial activity increased while as the *para *proton of aryl ring of pyrazole moiety is replaced with electron-withdrawing group, the antibacterial activity decreases.

Triazoles **4 **having electron-releasing substituents at *para *position of aryl ring of pyrazole moiety (**4a**, **4b **and **4c**) are more antifungal than triazoles containing electron-withdrawing groups (**4a**, **4e**, **4f **and **4g**) at the same position.

## Experimental

Melting points were taken on slides in an electrical apparatus Labindia visual melting range apparatus and are uncorrected. The IR spectra were obtained with a Buck Scientific IR M-500 spectrophotometer. The ^1^H NMR spectra were recorded on a Bruker (300 MHz) spectrometer using tetramethylsilane as an internal standard. All the compounds gave satisfactory analytical results (within 0.4% of the theoretical values). The starting material 4-formylpyrazoles **2 **were prepared by the literature method [32].

### 2-((3-Aryl-1-phenyl-1H-pyrazol-4-yl)methylene)-1-(pyridin-2-yl)hydrazines (3)

General procedure: To the ethanolic solution of 2-pyridylhydrazine (**1**, 0.01 mol) was added appropriate 4-formylpyrazole (**2**, 0.01 mol), and the solution was refluxed for 4-5 min. The solvent was evaporated in vacuo to half its volume and cooled to room temperature. The solid obtained was filtered and washed with ethanol.

### 2-((1,3-Diphenyl-1H-pyrazol-4-yl)methylene)-1-(pyridin-2-yl)hydrazine (3a)

Yield 96%, Mp 210°C, IR (ν_max_, KBr): 3190 cm^-1 ^(-NH str.); ^1^H NMR (DMSO-*d*_6_, 300 MHz): δ 6.71-6.74 (m, 1H), 7.18-7.21 (m, 1H, *J *= 9 Hz), 7.34-7.39 (m, 1H), 7.47-7.63 (m, 6H), 7.74-7.77 (m, 2H), 7.98-8.01 (m, 2H), 8.07-8.09 (d, 1H, *J *= 6 Hz), 8.17(s, 1H, N=CH), 8.93(s, 1H), 10.66 (bs, 1H, exchangeable with D_2_O); Anal. Calculated for C_21_H_17_N_5_: C 74.32, H 5.05, N 20.63; Found: C 74.35, H 5.04, N 20.64; ESI-MS *m/z*: 340.06 [M + 1]^+^.

### 2**-((1-Phenyl-3-p-tolyl-1H-pyrazol-4-yl)methylene)-1-(pyridin-2-yl)hydrazine (3b)**

Yield 94%, Mp 206°C, IR (ν_max_, KBr): 3194 cm^-1 ^(-NH str.); ^1^H NMR (DMSO-*d_6_*, 300 MHz): δ 2.40 (s, 3H), 6.73-6.74 (m, 1H), 7.20-7.23 (d, 1H, *J *= 9 Hz), 7.32-7.38 (m, 3H), 7.51-7.59 (d, 2H, *J *= 9 Hz), 7.62-7.65 (m, 3H), 7.98-8.09 (m, 3H), 8.15 (s, 1H), 8.93(s, 1H, N=CH), 10.69 (bs, 1H, exchangeable with D_2_O); Anal. Calculated for C_22_H_19_N_5_: C 74.77, H 5.42, N 19.82; Found: C 74.79, H 5.45, N 19.79; ESI-MS *m/z*: 354.12 [M + 1]^+^

### 2-((3-(4-Methoxyphenyl)-1-phenyl-1H-pyrazol-4-yl)methylene)-1-(pyridin-2-yl)hydrazine (3c)

Yield 89%, Mp 208°C, IR (ν_max_, KBr): 3189 cm^-1 ^(-NH str.); ^1^H NMR (DMSO-*d_6_*, 300 MHz): δ 3.84(s, 3H), 6.70-6.74 (m, 1H), 7.07-7.10 (d, 2H, *J *= 9 Hz), 7.19-7.22 (m, 1H), 7.45-7.38 (m, 1H), 7.51-7.64 (m, 3H), 7.67-7.70 (d, 2H, *J *= 9 Hz), 7.97-8.00 (m, 2H), 8.07-8.09 (d, 1H, *J *= 6 Hz), 8.14 (s, 1H, N=CH), 8.91(s, 1H), 10.67 (bs, 1H, exchangeable with D_2_O); Anal. Calculated for C_22_H_19_N_5_O: C 71.53, H 5.18, N 18.96; Found: C 71.53, H 5.20, N 18.92; ESI-MS *m/z*: 370.08 [M + 1]^+^.

### 2-((3-(4-Fluorophenyl)-1-phenyl-1H-pyrazol-4-yl)methylene)-1-(pyridin-2-yl)hydrazine (3d)

Yield 90%, Mp 224°C, IR (ν_max_, KBr): 3198 cm^-1 ^(-NH str.); ^1^H NMR (DMSO-*d_6_*, 300 MHz): δ 6.71-6.75 (m, 1H), 7.14-7.17 (m, 1H), 7.33-7.39 (m, 3H), 7.52-7.63 (m, 3H), 7.79-7.84 (d, 2H, *J *= 9 Hz), 7.97-8.00 (d, 2H, *J *= 9 Hz), 8.08-8.09 (d, 1H, *J *= 3 Hz), 8.15 (s, 1H, N=CH), 8.93 (s, 1H), 10.66 (bs, 1H, exchangeable with D_2_O); Anal. Calculated for C_21_H_16_FN_5_: C 70.58, H 4.51, N 19.60; Found: C 70.59, H 4.53, N 19.58; ESI-MS *m/z*: 358.10 [M + 1]^+^.

### 2-((3-(4-Chlorophenyl)-1-phenyl-1H-pyrazol-4-yl)methylene)-1-(pyridin-2-yl)hydrazine (3e)

Yield 90%, Mp 232°C, IR (ν_max_, KBr): 3200 cm^-1 ^(-NH str.); ^1^H NMR (DMSO-*d*_6_, 300 MHz): δ 6.70-6.74 (m, 1H), 7.13-7.15 (m, 1H), 7.34-7.39 (m, 1H), 7.52-7.62 (m, 5H), 7.79-7.82 (d, 2H, *J *= 9 Hz), 7.96-7.99 (d, 2H, *J *= 9 Hz), 8.07-8.09 (d, 1H, *J *= 6 Hz), 8.15 (s, 1H, N=CH), 8.91 (s, 1H), 10.64 (bs, 1H, exchangeable with D_2_O); Anal. Calculated for C_21_H_16_ClN_5_: C 67.47, H 4.31, N 18.73; Found: C 67.41, H 4.30, N 18.75; ESI-MS *m/z*: 374.10 [M + 1]^+^, 376.10 [M + 3]^+^.

### 2-((3-(4-Bromophenyl)-1-phenyl-1H-pyrazol-4-yl)methylene)-1-(pyridin-2-yl)hydrazine (3f)

Yield 91%, Mp 236°C, IR (ν_max_, in KBr): 3203 cm^-1 ^(-NH str.); ^1^H NMR (DMSO-*d_6_*, 300 MHz): δ 6.69-6.73 (m, 1H), 7.12-7.14 (m, 1H), 7.35-7.38 (m, 1H), 7.50-7.59 (m, 3H), 7.71 (m, 4H), 7.95-7.98 (d, 2H, *J *= 8.1 Hz), 8.06-8.07 (d, 1H, *J *= 4.5 Hz), 8.12 (s, 1H, N=CH), 8.93(s, 1H), 10.67 (bs, 1H, exchangeable with D_2_O); Anal. Calculated for C_21_H_16_BrN_5_: C 60.30, H 3.86, N 16.74; Found: C 60.28, H 3.83, N 16.77; ESI-MS *m/z*: 385.12 ([M + 1]^+^, 387.02 [M + 3]^+^.

### 2-((3-(4-Nitrophenyl)-1-phenyl-1H-pyrazol-4-yl)methylene)-1-(pyridin-2-yl)hydrazine (3g)

Yield 92%, Mp 234°C, IR (ν_max_, in KBr): 3195 cm^-1 ^(-NH str.) 1335, 1504 (-NO_2_); ^1^H NMR (DMSO-*d_6_*, 300 MHz): δ 6.72-6.76 (m, 1H), 7.12-7.15 (m, 1H), 7.38-7.42 (m, 1H), 7.54-7.63 (m, 3H), 8.00-8.03 (m, 2H, *J *= 9 Hz), 8.08-8.11 (m, 3H), 8.20 (s, 1H, N=CH), 8.36-8.39 (d, 2H, *J *= 9 Hz), 9.01 (s, 1H), 10.76 (bs, 1H, exchangeable with D_2_O); Anal. Calculated for C_21_H_16_N_6_O_2_: C 65.62, H 4.20, N 21.86; Found: C 65.64, H 4.26, N 21.85; ESI-MS *m/z*: 267.06 [M + 1]^+^.

### Synthesis of 3-(3-aryl-1-phenyl-1H-pyrazol-4-yl)-[1,2,4]triazolo[4,3-a]pyridines (4)

General procedure: To a suspension/solution of **3 **(0.010 mol) in dichloromethane (25 mL), IBD (0.011 mol) was added in small portions, and the reaction mixture was stirred overnight. Then, the solvent was evaporated on water bath. To the resulting residue was added ethanol (5-10 mL), and the mixture was warmed to obtain a clear solution. On cooling at room temperature, solid separated out was filtered and washed with cold alcohol to give pure fused 1,2,4-triazole derivatives **4a-g**.

### (1,3-Diphenyl-1H-pyrazol-4-yl)-[1,2,4]triazolo[4,3-a]pyridine (4a)

Yield 90%, Mp 162°C, IR (ν_max_, KBr): transparent in the region of -NH str.; ^1^H NMR (DMSO-*d_6_*, 300 MHz): δ 6.89-6.94 (m, 1H), 7.33-7.35 (m, 3H), 7.38-7.45 (m, 2H), 7.54-7.63 (m, 4H), 7.84-7.87 (s, 1H), 7.99-8.05 (m, 2H), 8.15-8.17 (d, 1H, *J *= 6 Hz), 9.16 (s, 1H); Anal. Calculated for C_21_H_15_N_5_: C 74.76, H 4.48, N 20.76; Found: C 74.77, H 4.45, N 20.73; ESI-MS *m/z*: 338.09 [M + 1]^+^.

### 3-(1-Phenyl-3-p-tolyl-1H-pyrazol-4-yl)-[1,2,4]triazolo[4,3-a]pyridine (4b)

Yield 88%, Mp 210°C, IR (ν_max_, KBr): transparent in the region of -NH str.; ^1^H NMR (DMSO-*d_6_*, 300 MHz): δ 2.28 (s, 3H) 6.89-6.93 (m, 1H), 7.13-7.16 (m, 2H), 7.38-7.44 (m, 4H), 7.57-7.62 (m, 2H), 7.80-7.84 (m, 1H), 8.01-8.03 (d, 2H, *J *= 6 Hz), 8.12-8.14 (d, 1H, *J *= 6 Hz), 9.13 (s, 1H); Anal. Calculated for C_22_H_17_N_5_: C 75.19, H 4.88, N 19.93; Found: C 75.18, H 4.86, N 19.96; ESI-MS *m/z*: 352.11 [M + 1]^+^.

### 3-(3-(4-Methoxyphenyl)-1-phenyl-1H-pyrazol-4-yl)-[1,2,4]triazolo[4,3-a]pyridine (4c)

Yield 82%, Mp 164°C, IR (ν_max_, KBr): transparent in the region of -NH str.; ^1^H NMR (DMSO-*d_6_*, 300 MHz): δ 3.73 (s, 3H) 6.89-6.94 (m, 3H), 7.36-7.40 (m, 2H), 7.47-7.49 (m, 2H), 7.56-7.61 (m, 2H), 7.80-7.87 (m, 1H), 7.95-8.02 (m, 2H), 8.14-8.16 (m, 1H), 9.12 (s, 1H); Anal. Calculated for C_22_H_17_N_5_O: C 71.92, H 4.66, N 19.06; Found: C 71.91, H 4.64, N 19.07; ESI-MS *m/z*: 368.06 [M + 1]^+^.

### 3-(3-(4-Fluorophenyl)-1-phenyl-1H-pyrazol-4-yl)-[1,2,4]triazolo[4,3-a]pyridine (4d)

Yield 85%, Mp 193°C, IR (ν_max_, KBr): transparent in the region of -NH st.; ^1^H NMR (DMSO-*d_6_*, 300 MHz): δ 6.92-6.97 (m, 1H), 7.17-7.23 (m, 2H), 7.39-7.44 (m, 2H), 7.57-7.68 (m, 4H), 7.85-7.88 (m, 1H), 8.02-8.05 (d, 2H, *J *= 9 Hz), 8.24-8.26 (d, 1H, *J *= 6 Hz), 9.19 (s, 1H); Anal. Calculated for C_21_H_14_FN_5_: C 70.98, H 3.97, N 19.71; Found: C 70.98, H 3.99, N 19.69; ESI-MS *m/z*: 356.08 [M + 1]^+^.

### 3-(3-(4-Chlorophenyl)-1-phenyl-1H-pyrazol-4-yl)-[1,2,4]triazolo[4,3-a]pyridine (4e)

Yield 89%, Mp 133°C, IR (ν_max_, KBr): transparent in the region of -NH str.; ^1^H NMR (DMSO-*d_6_*, 300 MHz): δ 6.93-6.98 (m, 1H), 7.41-7.45 (m, 4H), 7.57-7.64 (m, 4H), 7.85-7.88 (m, 1H), 8.02-8.04 (d, 2H, *J *= 6 Hz), 8.25-8.27 (d, 1H, *J *= 6 Hz), 9.19 (s, 1H); Anal. Calculated for C_21_H_14_ClN_5_: C 67.83, H 3.80, N 18.84; Found: C 67.81, H 3.84, N 18.80; ESI-MS *m/z*: 372.01 [M + 1]^+^, 374.06 [M + 3]^+^.

### 3-(3-(4-Bromophenyl)-1-phenyl-1H-pyrazol-4-yl)-[1,2,4]triazolo[4,3-a]pyridine (4f)

Yield 84%, Mp 140°C, IR (ν_max_, KBr): transparent in the region of -NH str.; ^1^H NMR (DMSO-*d_6_*, 300 MHz): δ 6.92-6.96 (m, 1H), 7.38-7.43 (m, 2H), 7.54-7.60 (m, 6H), 7.83-7.86 (m, 1H), 7.99-8.02 (d, 2H, *J *= 8.1 Hz), 8.23-8.26 (d, 1H, *J *= 6.9 Hz), 9.17 (s, 1H); Anal. Calculated for C_21_H_14_BrN_5_: C 60.59, H 3.39, N 16.82; Found: C 60.56, H 3.37, N 16.81; ESI-MS *m/z*: 416.04 [M + 1]^+^, 418.06 [M + 3]^+^.

### 3-(3-(4-Nitrophenyl)-1-phenyl-1H-pyrazol-4-yl)-[1,2,4]triazolo[4,3-a]pyridine (4g)

Yield 89%, Mp 242°C, IR (ν_max_, KBr): transparent in the region of -NH str.; ^1^H NMR (DMSO-*d_6_*, 300 MHz): δ 6.82-6.86 (m, 1H), 7.34-7.41 (m, 1H), 7.44-7.46 (m, 1H), 7.55-7.60 (m, 3H), 7.83-7.87 (m, 3H), 7.92-7.94 (d, 2H, *J *= 6 Hz), 8.14-8.17 (d, 2H, *J *= 9 Hz), 8.71 (s, 1H); Anal. Calculated for C_21_H_14_N_6_O_2_: C 65.96, H 3.69, N 21.98; Found: C 65.97, H 3.67, N 21.94; ESI-MS *m/z*: 383.10 [M + 1]^+^.

## Biological assay

### Test microorganisms

Total six microbial strains were selected on the basis of their clinical importance in causing diseases in humans. Two Gram-positive bacteria (*Staphylococcus aureus *MTCC 96 and *Bacillus subtilis *MTCC 121); two Gram-negative bacteria (*Escherichia coli *MTCC 1652 and *Pseudomonas aeruginosa *MTCC 741) and two fungi, *Aspergillus niger *and *A. flavus*, the ear pathogens isolated from the patients of Kurukshetra, were used in the present study for the evaluation of antimicrobial activity of the compounds [33]. All the cultures were procured from Microbial Type Culture Collection (MTCC), IMTECH, Chandigarh. The bacteria were subcultured on nutrient agar, whereas fungi on Sabouraud dextrose.

### *In vitro *antibacterial activity

The antibacterial activity of chemical compounds was evaluated by the agar well diffusion method. All the cultures were adjusted to 0.5 McFarland standards, which is visually comparable to a microbial suspension of approximately 1.5 × 10^8 ^cfu/mL. 20 mL of Mueller Hinton agar medium was poured into each Petri plate, and the agar plates were swabbed with 100 μL inocula of each test bacterium and kept for 15 min for adsorption. Using sterile cork borer of 8 mm diameter, wells were bored into the seeded agar plates, and these were loaded with a 100 μL volume with concentration of 2.0 mg/mL of each compound reconstituted in the dimethylsulphoxide (DMSO). All the plates were incubated at 37°C for 24 h. Antibacterial activity of each compound was evaluated by measuring the zone of growth inhibition against the test organisms with zone reader (Hi Antibiotic zone scale). The DMSO was used as a negative control, whereas ciprofloxacin was used as a positive control. This procedure was performed in three replicate plates for each organism [34].

## Determination of minimum inhibitory concentration (MIC)

The MIC is the lowest concentration of an antimicrobial compound that will inhibit the visible growth of a microorganism after overnight incubation. The MIC of all the synthesized compounds (**3 **and **4**) against bacterial strains was tested through a modified agar well-diffusion method [35]. In this method, a twofold serial dilution of each compound was prepared by first reconstituting the compound in DMSO followed by dilution in sterile, distilled water to achieve a decreasing concentration range of 256-0.5 μg/mL. A 100 μL volume of each dilution was introduced into wells (in triplicate) in the agar plates already seeded with 100 μL of standardized inoculum (10^6 ^cfu/mL) of the test microbial strain. All test plates were incubated aerobically at 37°C for 24 h and observed for the inhibition zones. The MIC, taken as the lowest concentration of the chemical compound that completely inhibited the growth of the microbe, showed by a clear zone of inhibition, was recorded for each test organism. Ciprofloxacin was used as positive control while DMSO as negative control.

### *In vitro *antifungal activity

The antifungal activity of the newly synthesized compounds was evaluated by poison food technique. The moulds were grown on Sabouraud dextrose agar (SDA) at 25°C for 7 days and used as inocula. 15 mL of molten SDA (45°C) was poisoned by the addition of 100 μL volume of each compound having concentration of 4.0 mg/mL, reconstituted in the DMSO, poured into a sterile Petri plate and allowed to solidify at room temperature. The solidified poisoned agar plates were inoculated at the centre with fungal plugs (8 mm diameter), obtained from the actively growing colony and incubated at 25°C for 7 days. The DMSO was used as the negative control, whereas fluconazole was used as the positive control. The experiments were performed in triplicates. Diameter of the fungal colonies was measured and expressed as percent mycelial inhibition determined by applying the following formula [36]:

d*c*, average diameter of fungal colony in negative control plates; d*t *average diameter of fungal colony in experimental plates.

## Abbreviations

DMSO: dimethylsulphoxide; IBD: iodobenzene diacetate; MIC: minimum inhibitory concentration; MTCC: microbial type culture collection; SDA: sabouraud dextrose agar.

## Competing interests

The authors declare that they have no competing interests.

## Supplementary Material

Additional file 1 **^1^H NMR spectra**. (3a): ^1^H NMR of *2-((1,3-Diphenyl-1H-pyrazol-4-yl)methylene)-1-(pyridin-2-yl)hydrazine*. Click here for file

Additional file 2 **^1^H NMR spectra**. (4a): ^1^H NMR of *(1,3-Diphenyl-1H-pyrazol-4-yl)-[1,2,4]triazolo[4,3-a]pyridine*. Click here for file

Additional file 3 **^1^H NMR spectra**. (3b): ^1^H NMR of 2*-((1-Phenyl-3-p-tolyl-1H-pyrazol-4-yl)methylene)-1-(pyridin-2-yl)hydrazine*. Click here for file

Additional file 4 **^1^H NMR spectra**. (3c): ^1^H NMR of *2-((3-(4-Methoxyphenyl)-1-phenyl-1H-pyrazol-4-yl)methylene)-1-(pyridin-2-yl)hydrazine*. Click here for file

Additional file 5 **^1^H NMR spectra**. (3d): ^1^H NMR of *2-((3-(4-Fluorophenyl)-1-phenyl-1H-pyrazol-4-yl)methylene)-1-(pyridin-2-yl)hydrazine*. Click here for file

Additional file 6 **^1^H NMR spectra**. (3e): ^1^H NMR of *2-((3-(4-Chlorophenyl)-1-phenyl-1H-pyrazol-4-yl)methylene)-1-(pyridin-2-yl)hydrazine*. Click here for file

Additional file 7 **^1^H NMR spectra**. (3f): ^1^H NMR of *2-((3-(4-Bromophenyl)-1-phenyl-1H-pyrazol-4-yl)methylene)-1-(pyridin-2-yl)hydrazine*. Click here for file

Additional file 8 **^1^H NMR spectra**. (3g): ^1^H NMR of *2-((3-(4-Nitrophenyl)-1-phenyl-1H-pyrazol-4-yl)methylene)-1-(pyridin-2-yl)hydrazine*. Click here for file

Additional file 9 **^1^H NMR spectra**. (4b): ^1^H NMR of *3-(1-Phenyl-3-p-tolyl-1H-pyrazol-4-yl)-[1,2,4]triazolo[4,3-a]pyridine*. Click here for file

Additional file 10 **^1^H NMR spectra**. (4c): ^1^H NMR of *3-(3-(4-Methoxyphenyl)-1-phenyl-1H-pyrazol-4-yl)-[1,2,4]triazolo[4,3-a]pyridine*. Click here for file

Additional file 11 **^1^H NMR spectra**. (4d): ^1^H NMR of *3-(3-(4-Fluorophenyl)-1-phenyl-1H-pyrazol-4-yl)-[1,2,4]triazolo[4,3-a]pyridine*. Click here for file

Additional file 12 **^1^H NMR spectra**. (4e): ^1^H NMR of *3-(3-(4-Chlorophenyl)-1-phenyl-1H-pyrazol-4-yl)-[1,2,4]triazolo[4,3-a]pyridine*. Click here for file

Additional file 13 **^1^H NMR spectra**. (4f): ^1^H NMR of *3-(3-(4-Bromophenyl)-1-phenyl-1H-pyrazol-4-yl)-[1,2,4]triazolo[4,3-a]pyridine*. Click here for file

Additional file 14 **^1^H NMR spectra**. (4g): ^1^H NMR of *3-(3-(4-Nitrophenyl)-1-phenyl-1H-pyrazol-4-yl)-[1,2,4]triazolo[4,3-a]pyridine*. Click here for file
