# Supplementary figures and images for: A facile iodine(III)-mediated synthesis of 3-(3-aryl-1-phenyl-1H-pyrazol-4-yl)-[1,2,4]triazolo[4,3-a]pyridines via oxidation of 2-((3-aryl-1-phenyl-1H-pyrazol-4-yl)methylene)-1-(pyridin-2-yl)hydrazines and their antimicrobial evaluations
Source: Org Med Chem Lett. 2011 Jul 18;1:1. doi: 10.1186/2191-2858-1-1 (PMC3279144; doi:10.1186/2191-2858-1-1)

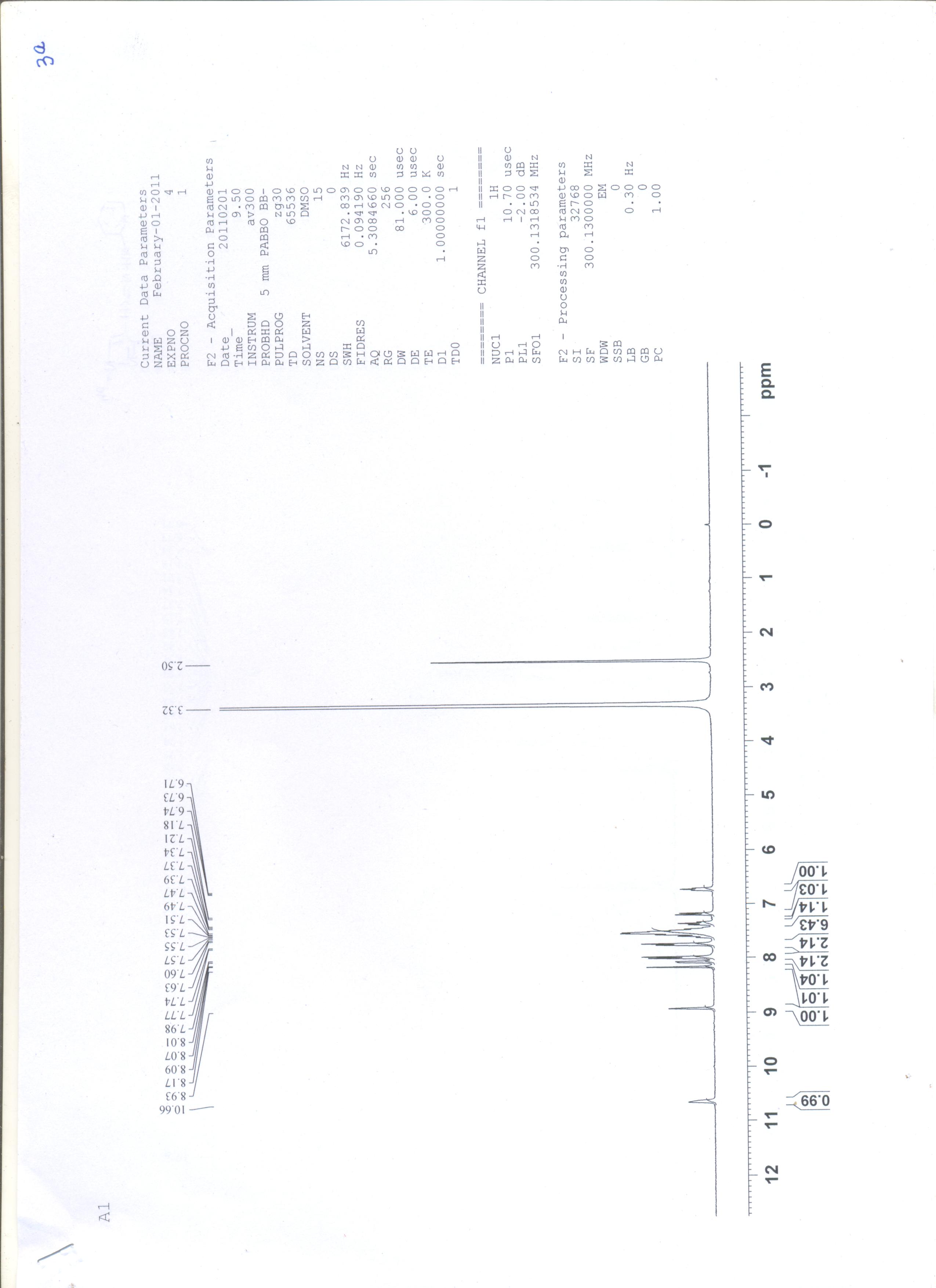

Supplement: Additional file 1 — 1H NMR spectra. (3a): 1H NMR of 2-((1,3-Diphenyl-1H-pyrazol-4-yl)methylene)-1-(pyridin-2-yl)hydrazine. [file 2191-2858-1-1-S1.JPEG]

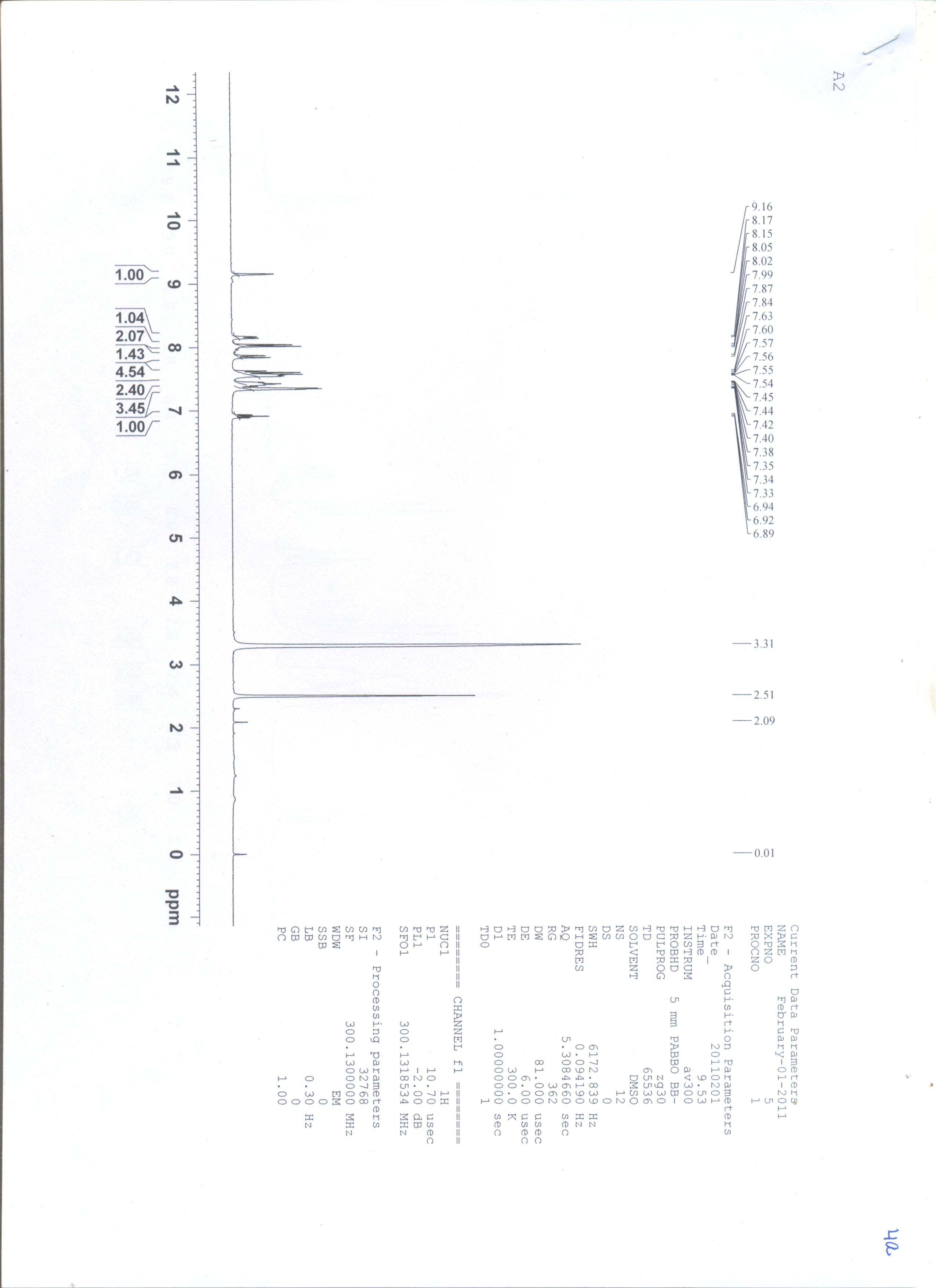

Supplement: Additional file 2 — 1H NMR spectra. (4a): 1H NMR of (1,3-Diphenyl-1H-pyrazol-4-yl)-[1,2,4]triazolo[4,3-a]pyridine. [file 2191-2858-1-1-S2.JPEG]

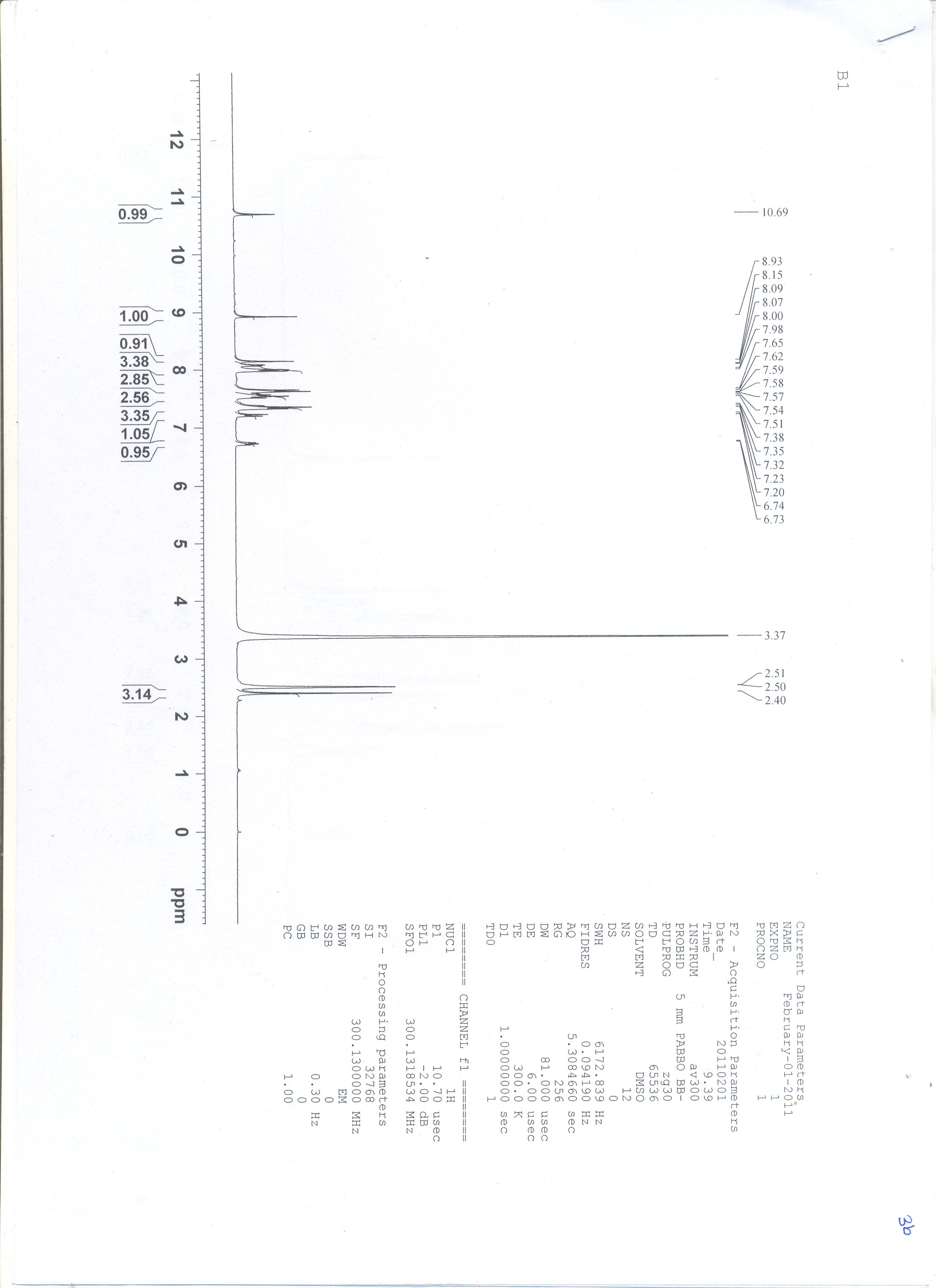

Supplement: Additional file 3 — 1H NMR spectra. (3b): 1H NMR of 2-((1-Phenyl-3-p-tolyl-1H-pyrazol-4-yl)methylene)-1-(pyridin-2-yl)hydrazine. [file 2191-2858-1-1-S3.JPEG]

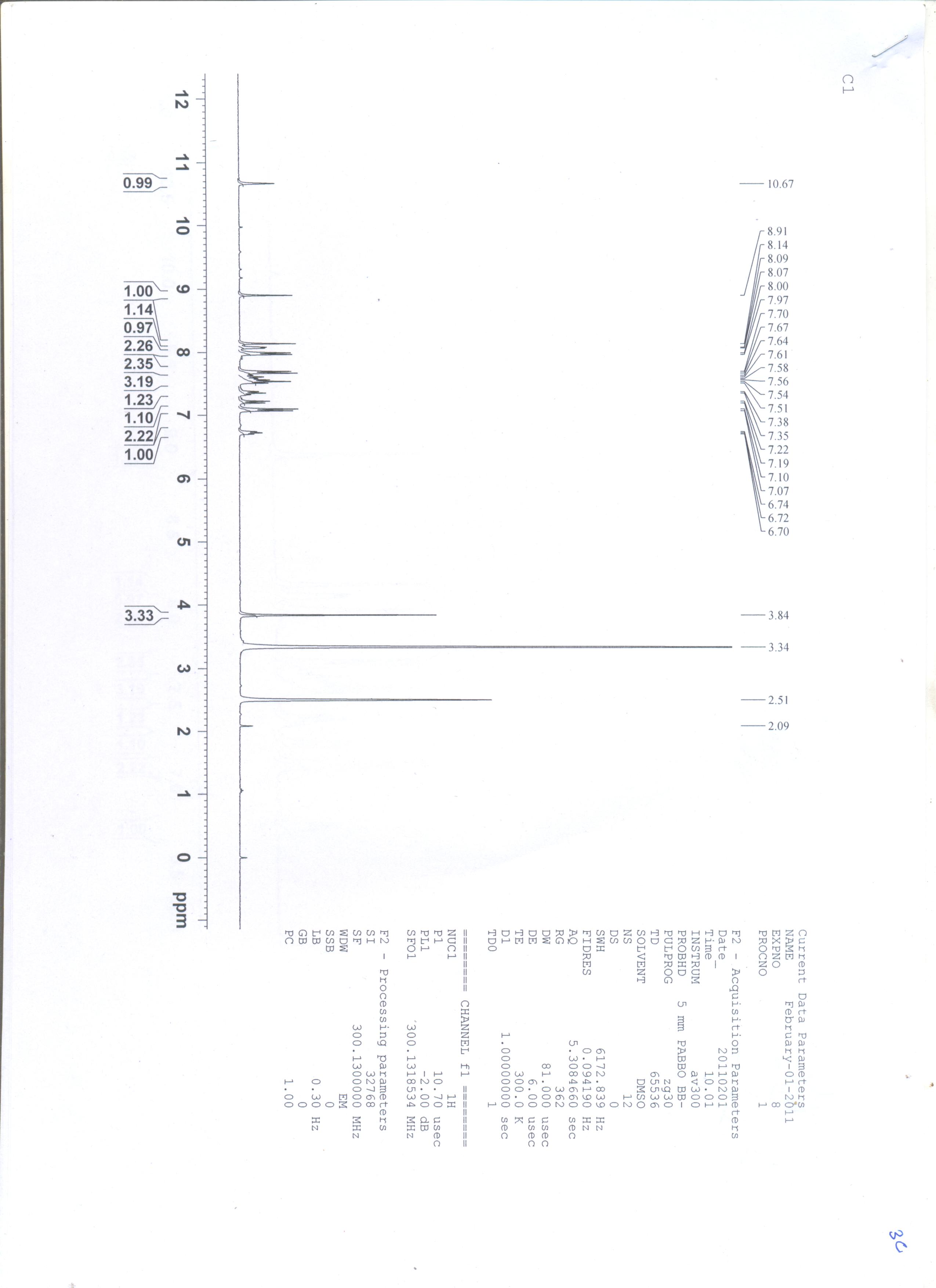

Supplement: Additional file 4 — 1H NMR spectra. (3c): 1H NMR of 2-((3-(4-Methoxyphenyl)-1-phenyl-1H-pyrazol-4-yl)methylene)-1-(pyridin-2-yl)hydrazine. [file 2191-2858-1-1-S4.JPEG]

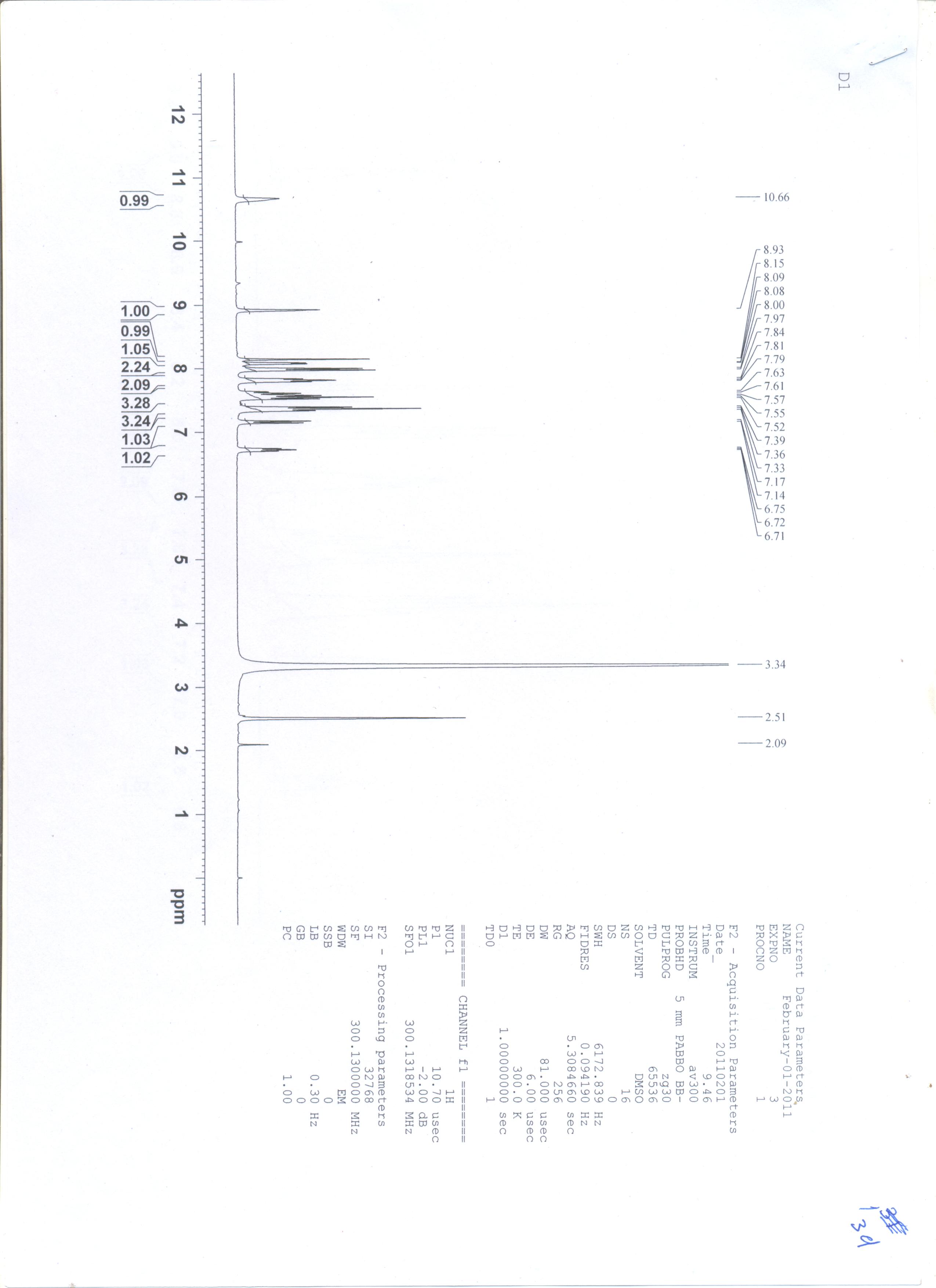

Supplement: Additional file 5 — 1H NMR spectra. (3d): 1H NMR of 2-((3-(4-Fluorophenyl)-1-phenyl-1H-pyrazol-4-yl)methylene)-1-(pyridin-2-yl)hydrazine. [file 2191-2858-1-1-S5.JPEG]

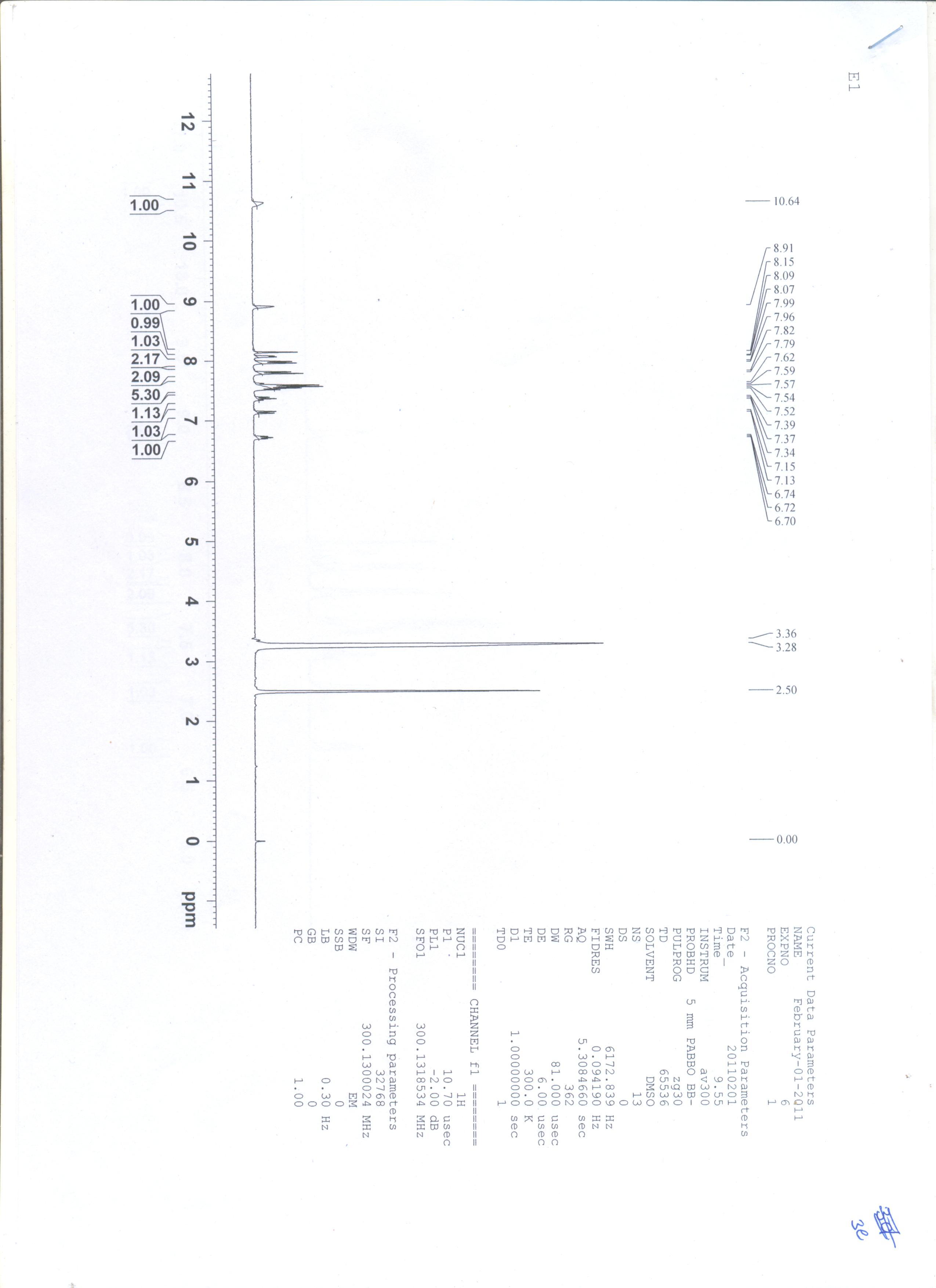

Supplement: Additional file 6 — 1H NMR spectra. (3e): 1H NMR of 2-((3-(4-Chlorophenyl)-1-phenyl-1H-pyrazol-4-yl)methylene)-1-(pyridin-2-yl)hydrazine. [file 2191-2858-1-1-S6.JPEG]

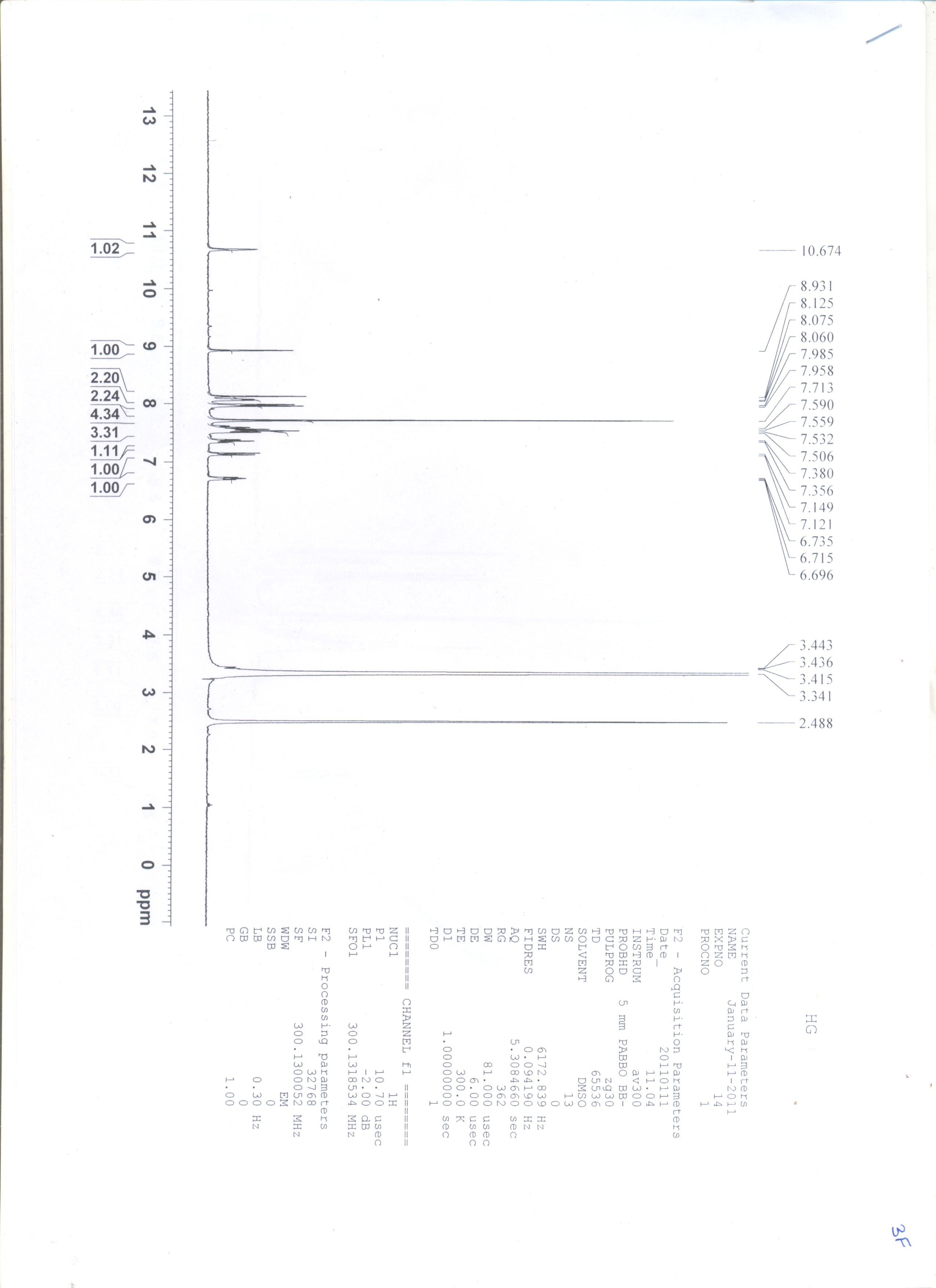

Supplement: Additional file 7 — 1H NMR spectra. (3f): 1H NMR of 2-((3-(4-Bromophenyl)-1-phenyl-1H-pyrazol-4-yl)methylene)-1-(pyridin-2-yl)hydrazine. [file 2191-2858-1-1-S7.JPEG]

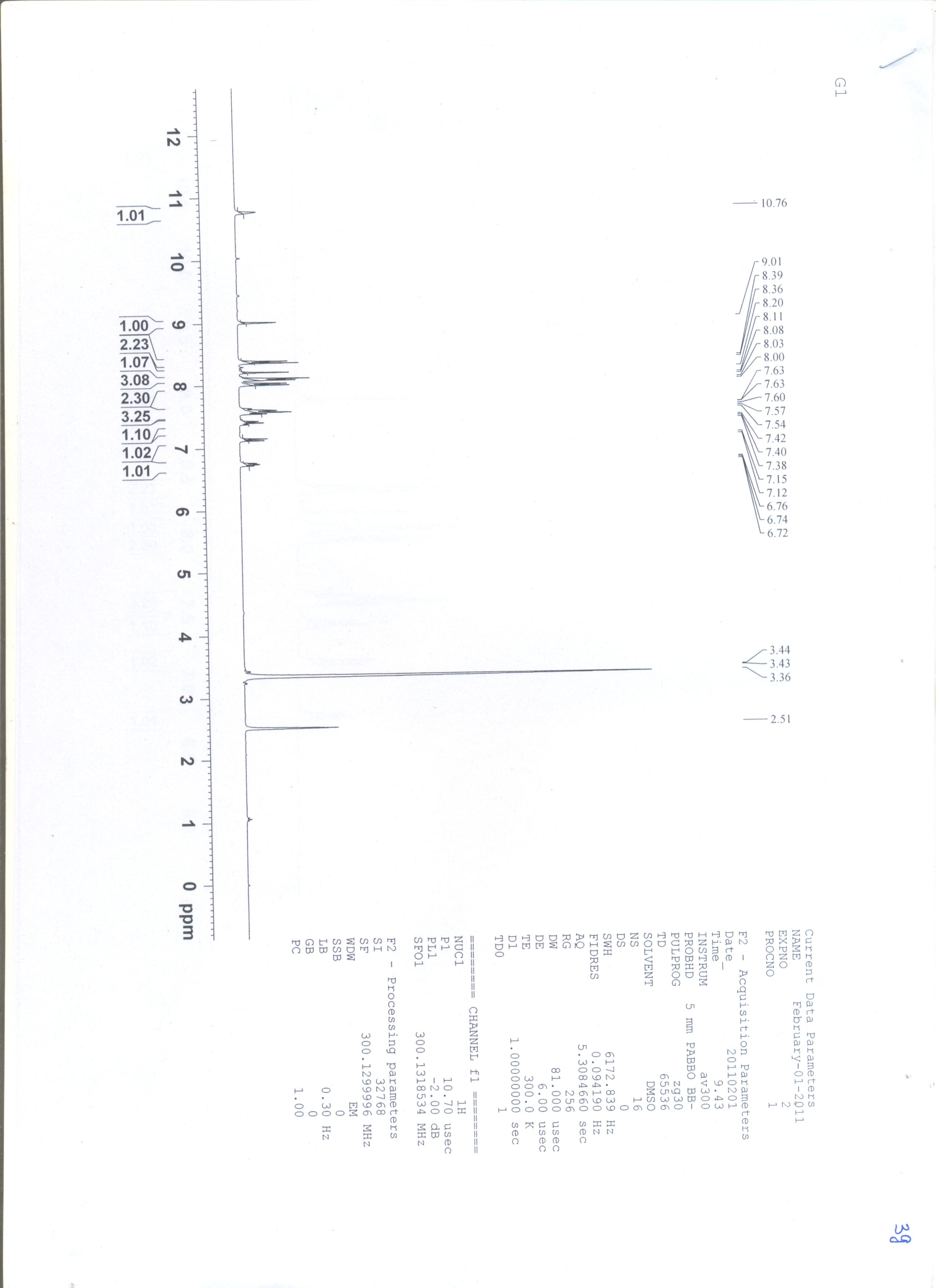

Supplement: Additional file 8 — 1H NMR spectra. (3g): 1H NMR of 2-((3-(4-Nitrophenyl)-1-phenyl-1H-pyrazol-4-yl)methylene)-1-(pyridin-2-yl)hydrazine. [file 2191-2858-1-1-S8.JPEG]

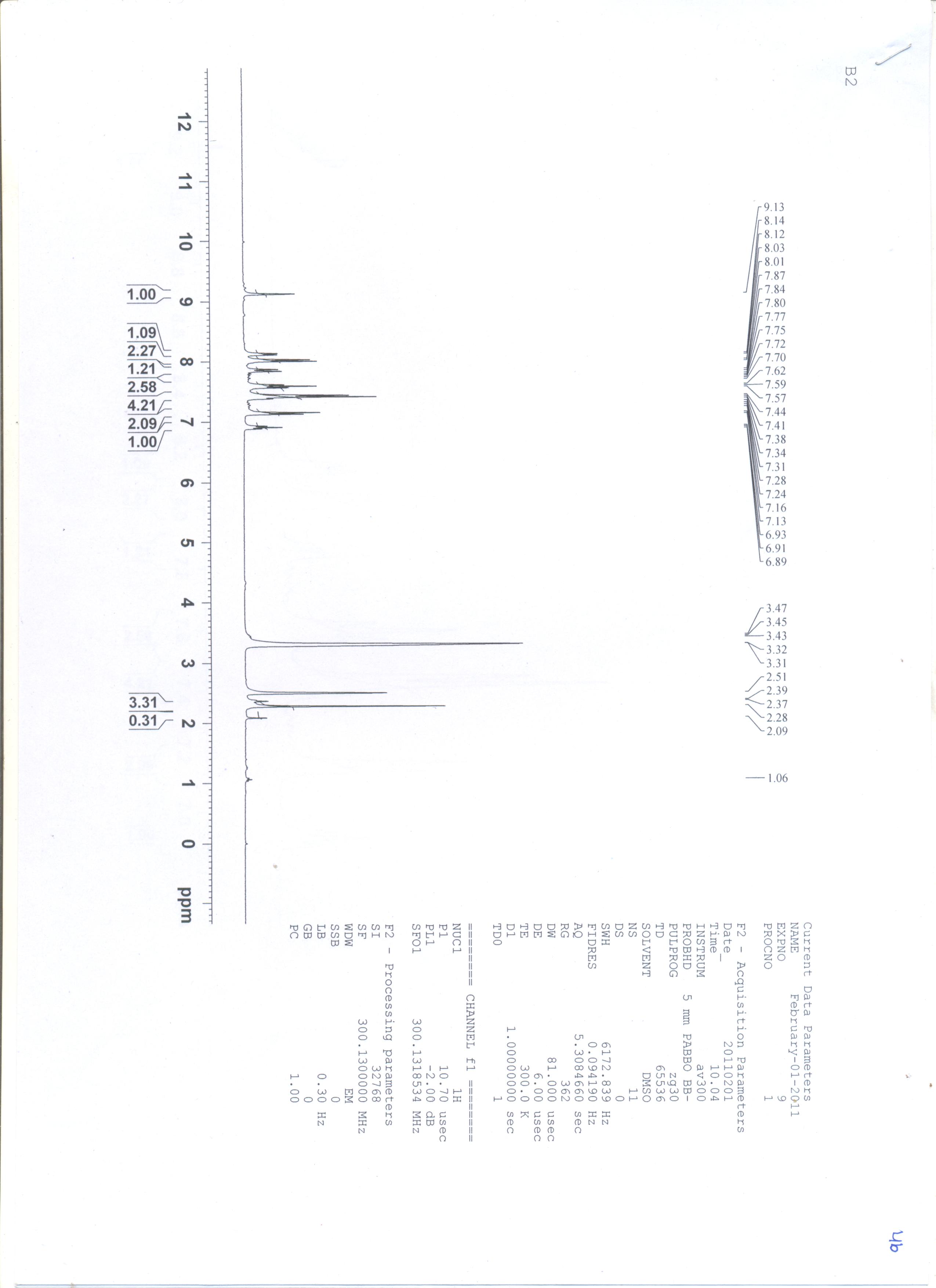

Supplement: Additional file 9 — 1H NMR spectra. (4b): 1H NMR of 3-(1-Phenyl-3-p-tolyl-1H-pyrazol-4-yl)-[1,2,4]triazolo[4,3-a]pyridine. [file 2191-2858-1-1-S9.JPEG]

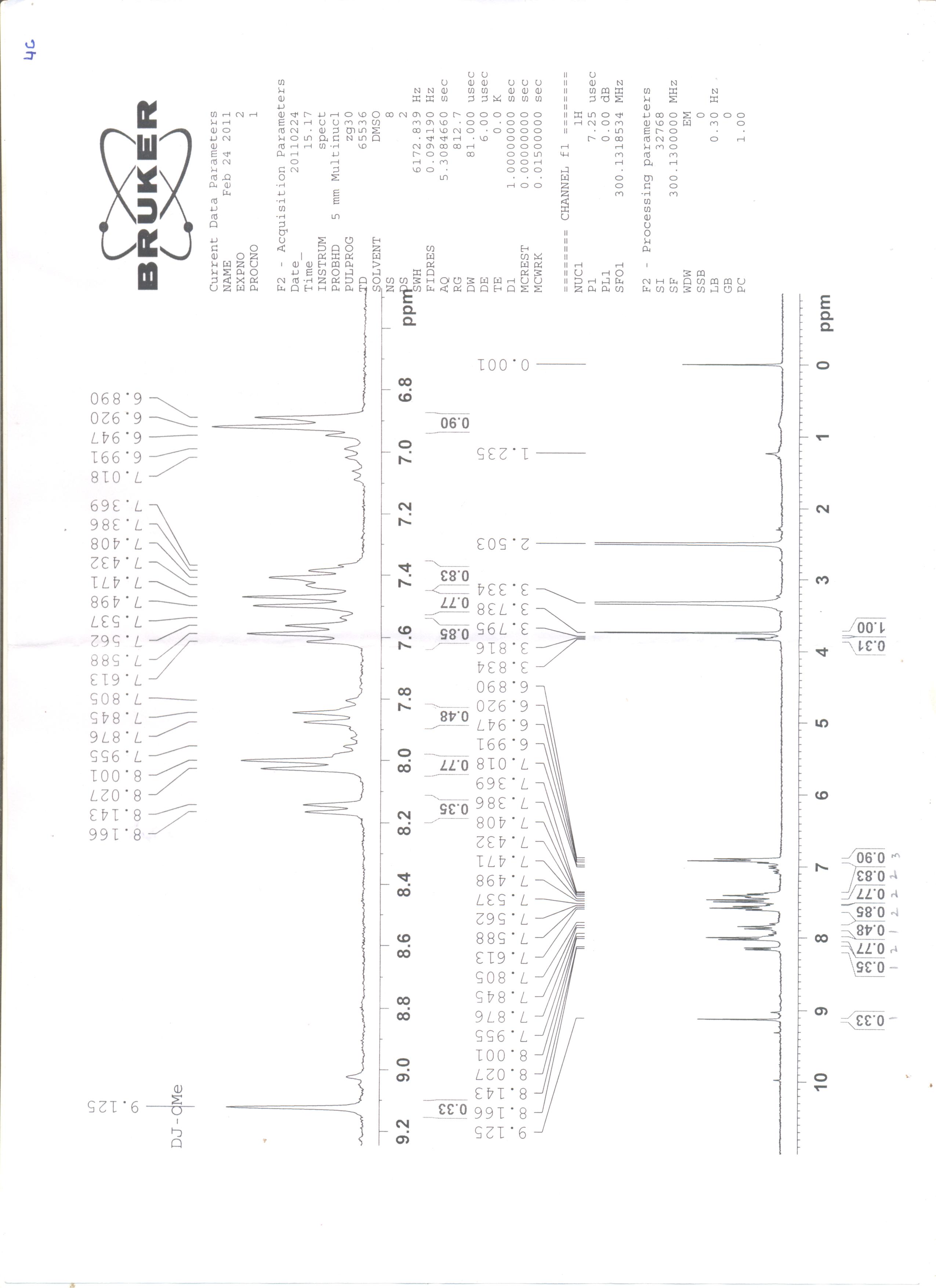

Supplement: Additional file 10 — 1H NMR spectra. (4c): 1H NMR of 3-(3-(4-Methoxyphenyl)-1-phenyl-1H-pyrazol-4-yl)-[1,2,4]triazolo[4,3-a]pyridine. [file 2191-2858-1-1-S10.JPEG]

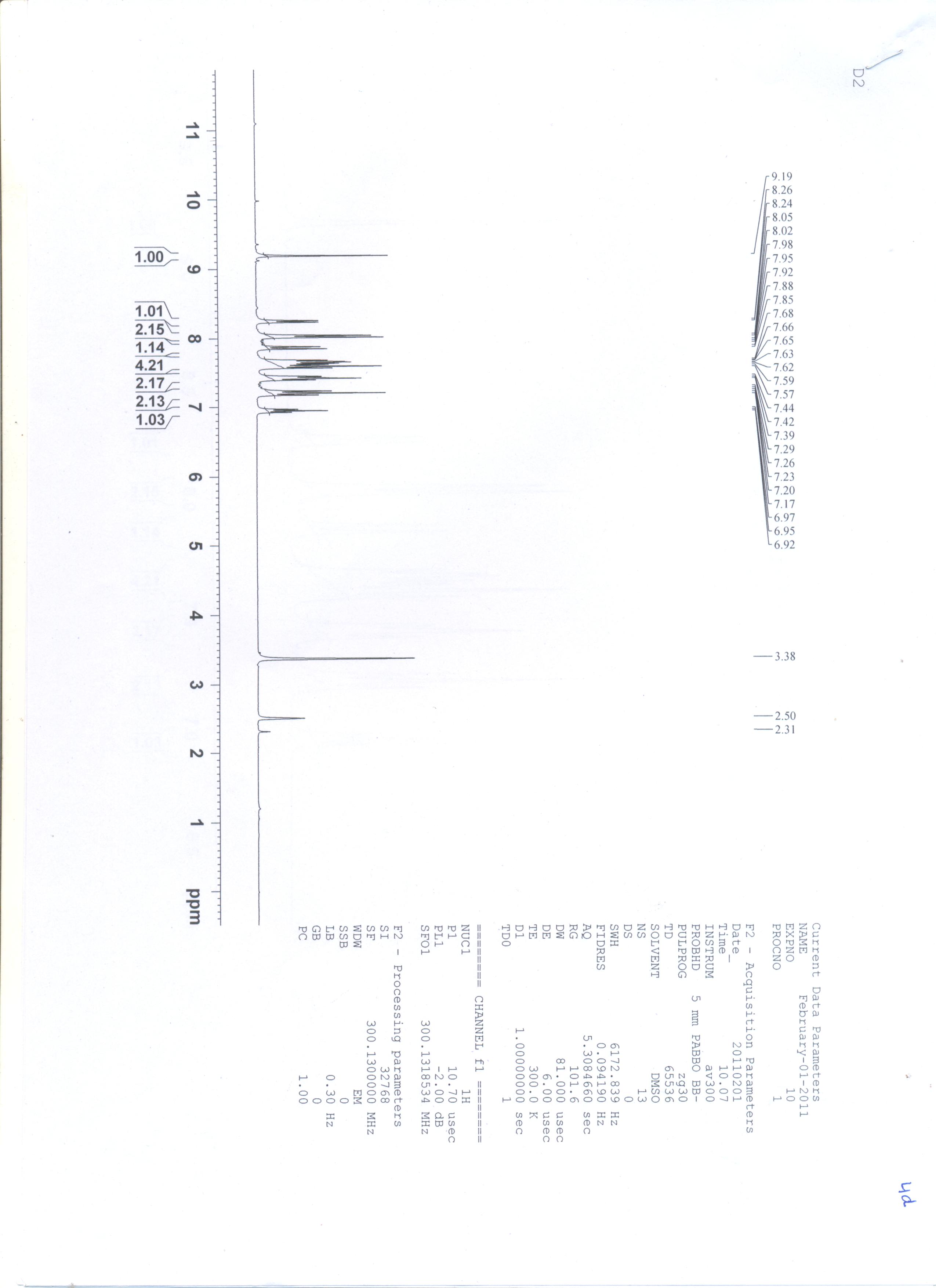

Supplement: Additional file 11 — 1H NMR spectra. (4d): 1H NMR of 3-(3-(4-Fluorophenyl)-1-phenyl-1H-pyrazol-4-yl)-[1,2,4]triazolo[4,3-a]pyridine. [file 2191-2858-1-1-S11.JPEG]

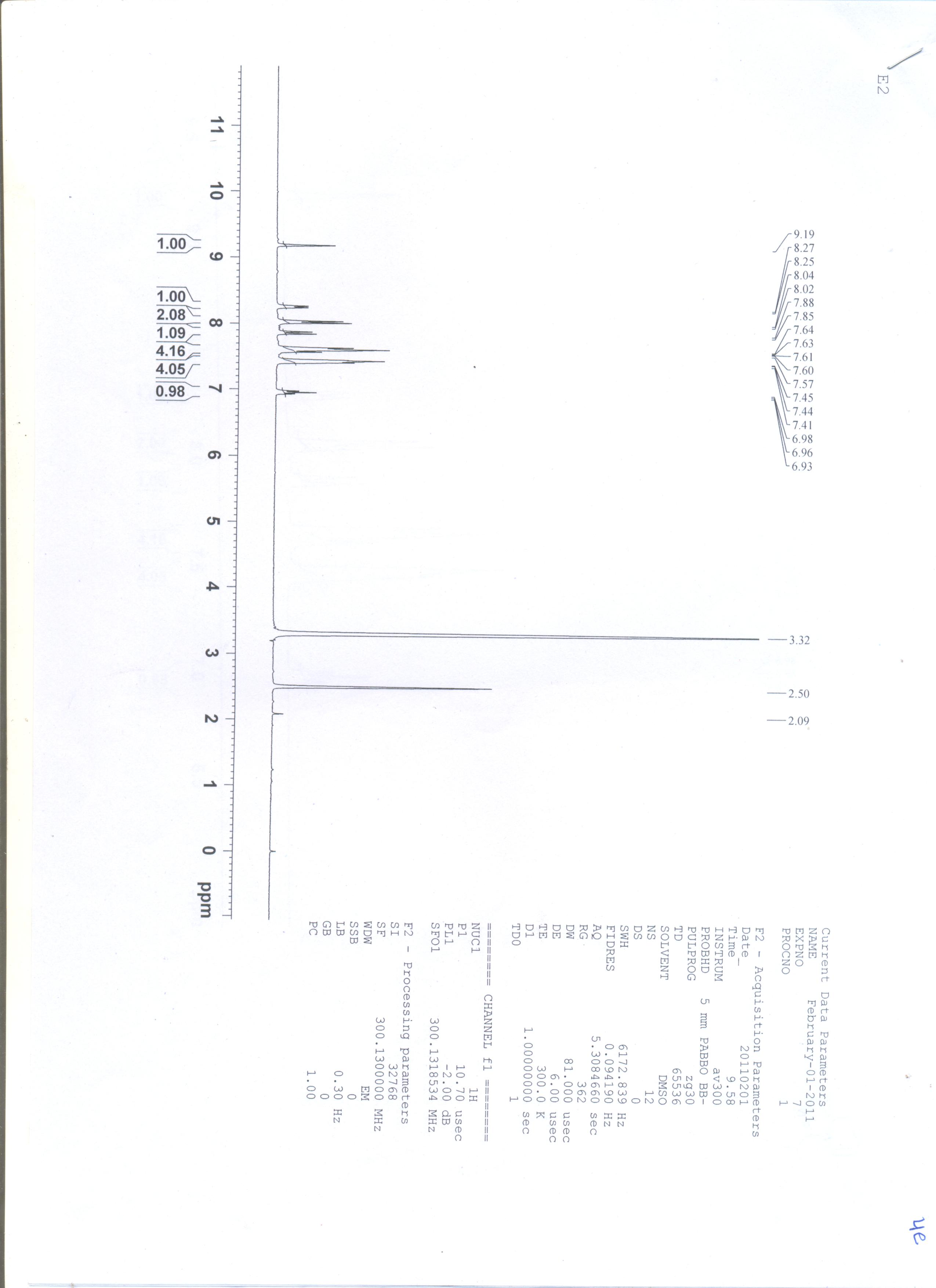

Supplement: Additional file 12 — 1H NMR spectra. (4e): 1H NMR of 3-(3-(4-Chlorophenyl)-1-phenyl-1H-pyrazol-4-yl)-[1,2,4]triazolo[4,3-a]pyridine. [file 2191-2858-1-1-S12.JPEG]

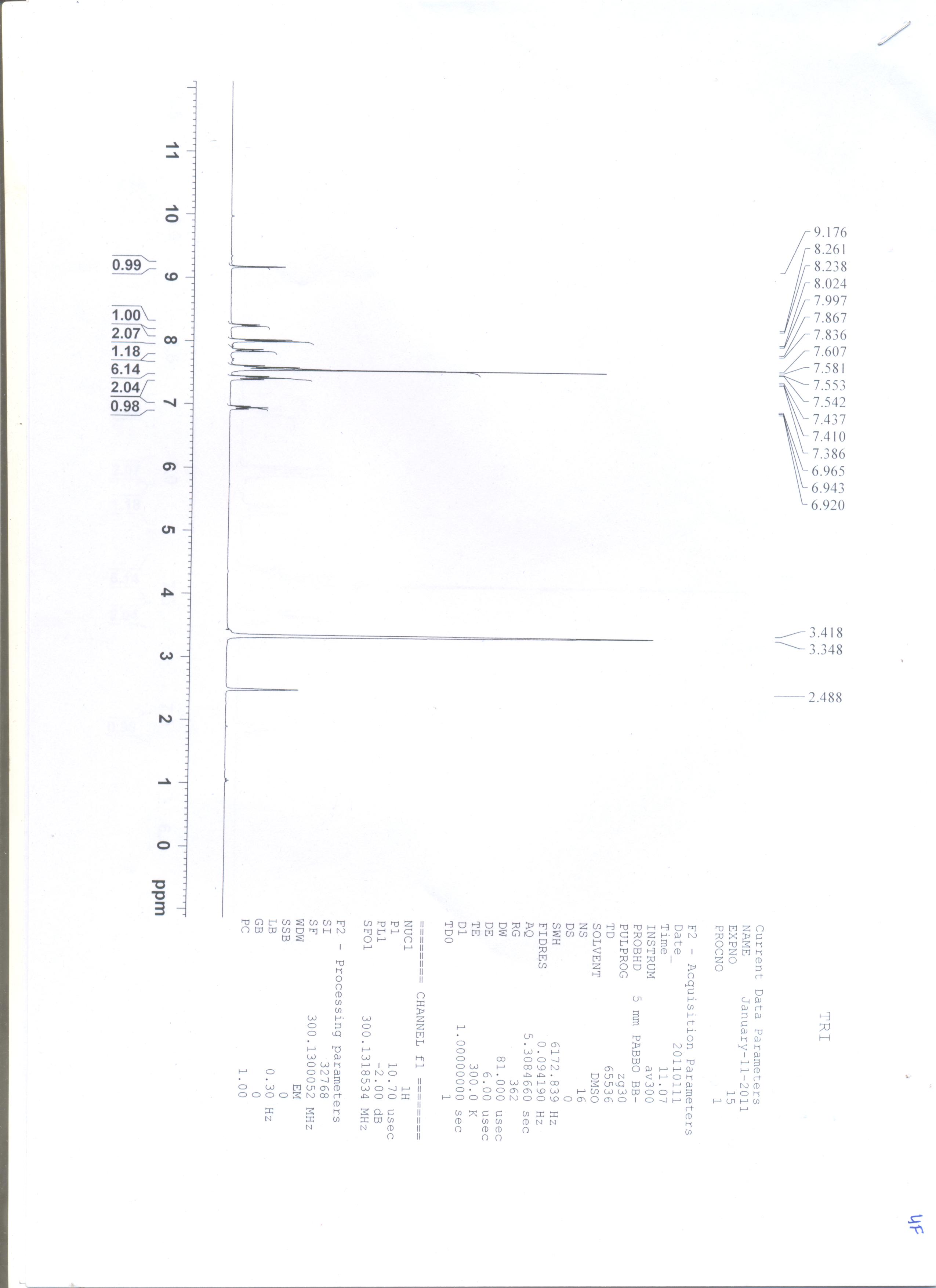

Supplement: Additional file 13 — 1H NMR spectra. (4f): 1H NMR of 3-(3-(4-Bromophenyl)-1-phenyl-1H-pyrazol-4-yl)-[1,2,4]triazolo[4,3-a]pyridine. [file 2191-2858-1-1-S13.JPEG]

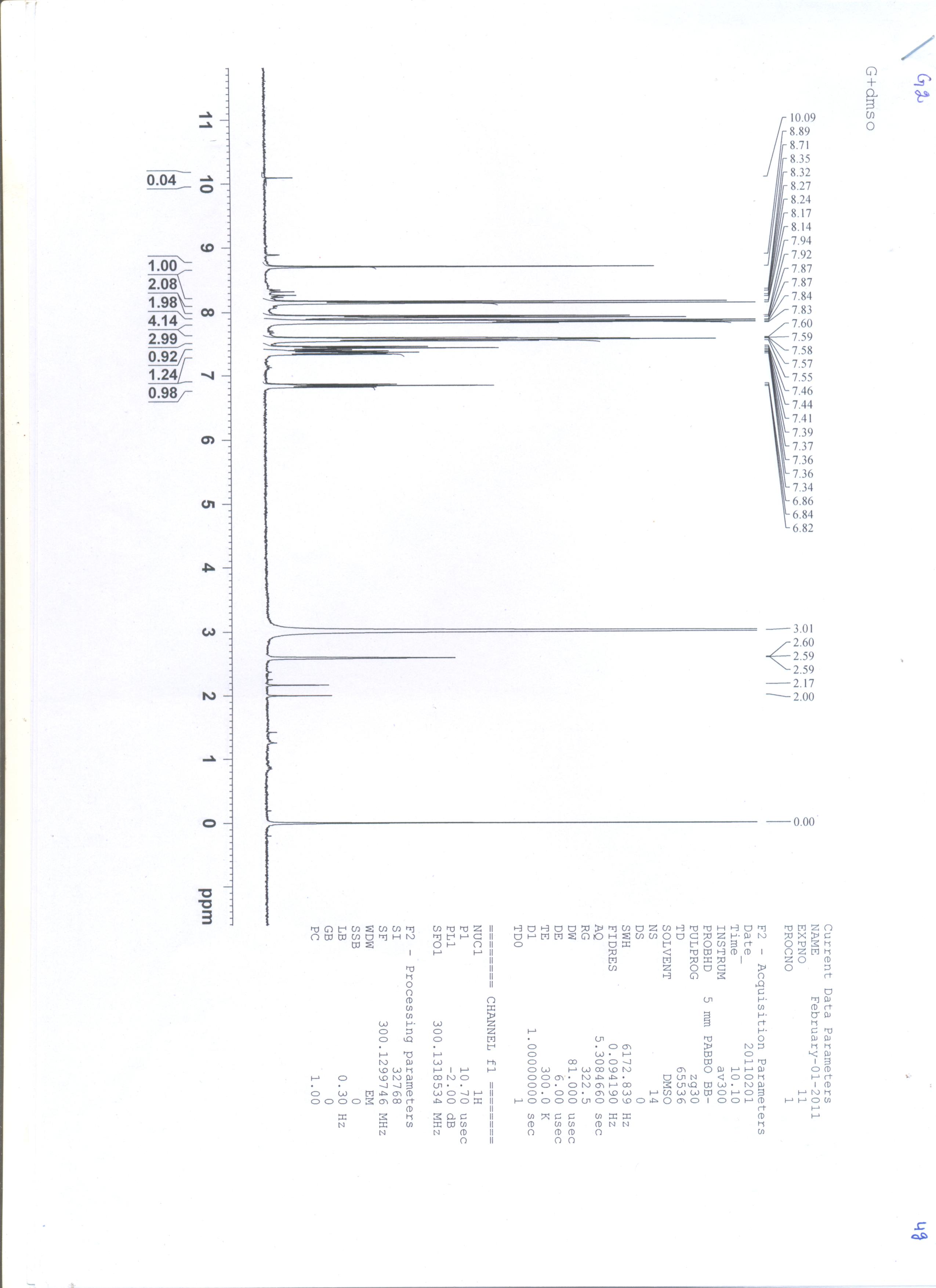

Supplement: Additional file 14 — 1H NMR spectra. (4g): 1H NMR of 3-(3-(4-Nitrophenyl)-1-phenyl-1H-pyrazol-4-yl)-[1,2,4]triazolo[4,3-a]pyridine. [file 2191-2858-1-1-S14.JPEG]
